# Impact of Insecticide-Treated Nets on *Plasmodium falciparum* Infection Rates: A Meta-Analysis

**DOI:** 10.3390/tropicalmed11050137

**Published:** 2026-05-18

**Authors:** Nevra Karaca Biçakçi, Ayşe Çalmaz, Merve Ayyildiz Akin, Ching Siang Tan, Jayanthi Barasarathi, Babatunji E. Oyinloye, Annaev Umidjon, Kuvonchbek Egamberdiyev, Intizor Avazmetova

**Affiliations:** 1 Department of Nursing, Faculty of Health Sciences, Kafkas University, Kars 36100, Türkiye; 2 İskilip Vocational School, Hitit University, Çorum 19030, Türkiye; 3 Department of Biostatistics, Faculty of Veterinary Medicine, Kafkas University, Kars 36100, Türkiye; 4 School of Pharmacy, KPJ Healthcare University, Nilai 71800, Malaysia; 5 Faculty of Health & Life Sciences (FHLS), INTI International University, Nilai 71800, Malaysia; 6 Institute for Drug Research and Development, Bogoro Research Centre, Afe Babalola University, Ado-Ekiti 360001, Nigeria; 7 Biotechnology and Structural Biology (BSB) Group, Department of Biochemistry and Microbiology, University of Zululand, KwaDlangezwa 3886, South Africa; 8 Phytomedicine, Biochemical Toxicology and Biotechnology Research Laboratories, Department of Biochemistry, College of Sciences, Afe Babalola University, Ado-Ekiti 360001, Nigeria; 9 Department of Natural Sciences, Termez University of Economics and Service, Termez 190100, Uzbekistan; 10 Department of Clinical Sciences, Ma’mun University, Urgench 220100, Uzbekistan; 11 Department of Biology, Urgench State University, Urgench 220100, Uzbekistan

**Keywords:** insecticide-treated nets, long-lasting insecticidal nets, *Plasmodium falciparum*, malaria prevention, meta-analysis, infection prevalence

## Abstract

Insecticide-treated nets (ITNs), particularly long-lasting insecticide-treated nets (LLINs), are important for malaria control; however, the rise of insecticide resistance, behavioral adaptations in mosquito vectors, and diminishing net durability may affect their efficacy. The objective of this systematic literature review and meta-analysis to synthesize recent epidemiological evidence (2021–2025) on the correlation between ITN/LLIN use and *Plasmodium falciparum* infection prevalence and to explore sources of heterogeneity across populations, settings, and type of nets. Searches across PubMed, Scopus, and Web of Science yielded 3151 records, of which 10 met the inclusion criteria. Two separate meta-analyses were performed for crude and adjusted effect estimates using random-effects models. The crude-effects meta-analysis included six studies comprising 7466 participants and yielded a pooled odds ratio of 0.67 (95% CI: 0.42–1.07; *p* = 0.078), indicating no statistically significant association between ITN/LLIN use and *P. falciparum* infection. Significant heterogeneity was observed (I^2^ = 79.8%), which was partially explained by population type (59.3%) and study design (36.1%). Subgroup analysis revealed comparable infection prevalence–based associations for ITNs (OR = 0.72) and LLINs (OR = 0.59) use. Assessment of publication bias indicated slight asymmetry; however, the trim-and-fill adjustment did not significantly change the conclusions. The adjusted-effects meta-analysis, comprising nine studies, yielded a non-significant pooled effect (aOR = 0.88; 95% CI: 0.42–1.86; *p* = 0.71) with substantial heterogeneity (I^2^ = 88.7%). Meta-regression analyses indicated that effect estimates varied by population group and country, with statistically significant modifiers observed for children under five years (*p* = 0.0098) and for studies conducted in Uganda (*p* = 0.0378). The type of net contributed to variation between studies, with insecticide-treated nets (ITNs) exhibiting lower pooled odds than long-lasting insecticide-treated nets (LLINs) (*p* = 0.0415). Overall, this meta-analysis found no conclusive evidence of a statistically significant association between ITN/LLIN use and *P. falciparum* infection in contemporary epidemiological studies. The substantial heterogeneity across settings underscores the context-dependent nature of observed associations and highlights the need for integrated vector-control strategies and continued evaluation of net technologies under conditions of increasing resistance.

## 1. Introduction

Malaria remains a leading cause of morbidity and mortality in many low- and middle-income countries, with Plasmodium falciparum responsible for the most severe manifestations of disease, particularly in sub-Saharan Africa. While significant gains in reducing malaria cases and deaths were achieved after 2000, there are indications that these gains have begun to slow or even reverse. Over the last 10 years, cases and deaths have started to increase globally for many reasons, including health system disruptions, long-standing social and economic inequalities, and inadequate funding for control programs [1,2]. The elimination of malaria transmission has been facilitated mainly by vector control interventions, notably insecticide-treated nets (ITNs), long-lasting insecticidal nets (LLINs), and indoor residual spraying (IRS); ITNs have contributed the most to the global reduction in case numbers [3,4]. Nevertheless, emerging insecticide resistance in mosquitoes, shifts in mosquito biting behavior, and practical difficulties have raised questions about whether these tools alone will achieve or maintain malaria elimination endpoints [5,6].

Many studies have demonstrated the protective effects of ITNs and LLINs against *Plasmodium falciparum* infections. Previous reviews have demonstrated that ITNs reduce the occurrence of malaria in the community when compared to no nets or untreated nets [3], and meta-regression analyses indicate that LLINs could be more effective than ITNs, with a reduction in malaria risk of over 50% [7]. Surveillance at the community and individual levels also suggests that high community coverage reinforces individual protection, underscoring the value of the broad availability and continuous use of nets [4]. Recently conducted cluster-randomized trials and implementation studies also indicate additional benefits of next-generation LLINs with synergists or dual active ingredients in areas of very high pyrethroid resistance, albeit with different efficacy [8,9]. There is evidence from a few low-transmission settings that ITNs and IRS are complementary when used together [10]. However, field studies suggest several challenges. A large proportion of these nets experience a loss of physical strength and killing capacity within 1–2 years [11]. Mosquito behavior is also evolving, with more outdoor and early-evening biting, which can further reduce the effectiveness of nets [12,13]. Socioeconomic and geographic disparities in ITN and IRS coverage also constrain this effect [14]. In some areas, LLINs alone or in combination with IRS have little impact on infection rates [15], and large-scale demographic studies have shown that upscaling ITN coverage has not always translated into fewer malaria cases despite significantly reduced mortality [2].

Together, these results suggest that the effectiveness of ITNs and LLINs against *P. falciparum* infection is driven by a complex and dynamic interplay among multiple factors, including insecticide resistance, net durability, mosquito behavior, program delivery, and broader social inequalities [5,6]. Many early trials supporting ITN use were conducted before pyrethroid resistance spread, and subsequent studies varied widely in design, population characteristics, settings, and outcome measures. These include assessments of universal coverage efforts, community-based net use, and integrated control, such as ITN combined with IRS [3,4,9,10]. In light of these differences and the complexities related to resistance, outdoor biting, and waning net effect over time [8,11,12,15], there is a need for an updated synthesis that directly estimates the current efficacy of ITN or LLINs against *P. falciparum* infection prevalence. Accordingly, the objective of this systematic review and meta-analysis was to quantify the association between ITN/LLIN use and *P. falciparum* infection outcomes using recent epidemiological evidence (2021–2025), and to explore sources of heterogeneity across populations, settings, and net types. This synthesis aims to provide policymakers and program implementers with updated, context-specific evidence to inform rational vector control strategies in an era of increasing resistance and slowing progress.

## 2. Materials and Methods

### 2.1. Search Strategy

A systematic literature search was conducted according to the Preferred Reporting Items for Systematic Reviews and Meta-Analyses (PRISMA) to identify studies investigating the effects of ITNs/LLINs on outcomes of *P. falciparum* infection prevalence [16]. The search strategy included appropriate combinations of controlled vocabulary terms and keywords to ensure the broadest coverage of the epidemiological literature on malaria prevention. A PubMed, Scopus, and Web of Science search was conducted using database-specific Boolean strings for insecticide-treated nets, *P. falciparum*, and infection-related epidemiological outcomes (including prevalence and incidence). The final search strings are given below:

PubMed: (“Insecticide-Treated Bednets”[Mesh] OR “Insecticide Treated Nets” OR “Insecticide-Treated Nets” OR ITN OR ITNs OR “Long-Lasting Insecticidal Nets” OR LLIN OR LLINs OR “bed nets” OR “mosquito nets”) AND (“Malaria, Falciparum”[Mesh] OR “Plasmodium falciparum” OR “falciparum malaria”) AND (infection* OR prevalence OR incidence OR “infection rate”).

SCOPUS: TITLE-ABS-KEY ((“insecticide-treated net*” OR “insecticide treated bed net*” OR ITN OR ITNs OR “long-lasting insecticidal net*” OR LLIN OR LLINs OR “mosquito net*” OR “bed net*”) AND (“Plasmodium falciparum” OR “falciparum malaria”) AND (infection* OR “infection rate*” OR prevalence OR incidence)).

WOS: TS = ((“insecticide-treated net*” OR “insecticide treated bed net*” OR ITN OR ITNs OR “long-lasting insecticidal net*” OR LLIN OR LLINs OR “mosquito net*” OR “bed net*”) AND (“Plasmodium falciparum” OR “falciparum malaria”) AND (infection* OR “infection rate*” OR prevalence OR incidence)).

The search included peer-reviewed, English-language studies and was limited to studies available from 2021 to 2025. All retrieved records were uploaded to EndNote, and duplicates were removed following recommended procedures [17].

### 2.2. Screening

A multi-stage screening was conducted on all retrieved records in line with the PRISMA 2020 guidelines for a systematic and transparent study selection process. After removing duplicates, the records were initially screened by title to identify studies on insecticide-treated nets and *Plasmodium falciparum* infection-related epidemiological outcomes. Studies selected in the title screening underwent abstract level screening for ITN/LLIN exposure, *P. falciparum* infection status outcomes (including prevalence- or incidence-based measures), and human epidemiological research. Full texts of eligible abstracts were screened to determine whether they reported relevant exposure definitions, species-specific infection outcomes, accepted human study designs, and quantitative data suitable for meta-analysis. Screening decisions were made systematically and documented at each stage, resulting in a final set of studies to be incorporated into quantitative synthesis. Title/abstract screening and full-text eligibility assessment were conducted independently by two reviewers using the predefined criteria. Discrepancies were resolved through discussion and consensus.

### 2.3. Inclusion Criteria

Studies were included if they were peer-reviewed, published in English from 2021 to 2025, and examined insecticide-treated nets (ITN) or long-lasting insecticide-treated nets (LLIN) as interventions or exposures. Eligible studies had to include human epidemiological outcomes related to *P. falciparum* (infection status, prevalence, incidence, parasitemia, or transmission indicators). Only epidemiological study designs, such as RCTs, cluster RCTs, cohort studies, case–control studies, cross–sectional surveys, and human longitudinal surveillance studies, were included. Furthermore, studies were required to report quantitative data that could be extracted for effect size estimation (raw counts of infections by ITN/LLIN use, stratified prevalence or incidence values, and reported ORs or RRs). “Mosquito nets” or “bed nets” studies were considered only if specifically reported to be insecticide-treated.

### 2.4. Exclusion Criteria

Studies were excluded if they did not report outcomes specific to *P. falciparum*, did not assess ITNs or LLINs as the exposure of interest, and those focused on interventions other than ITNs such as indoor residual spraying, vaccination, antimalarial drugs, larviciding, and repellents. Studies restricted to non-falciparum malaria, non-human studies, or studies of vectors only; molecular or mechanistic studies that did not relate directly to human infection outcomes; or non-primary research such as reviews, commentaries, perspectives, modelling analyses, and routine surveillance, were excluded. Moreover, any study without precise quantitative data available for the meta-analysis was excluded from the quantitative synthesis.

### 2.5. Data Extraction

Data extraction was performed in two stages using a standard template to ensure consistency among studies. The study ID (author and publication year), country, epidemiological study design used (RCT, cluster-RCT, cohort, case–control, or cross-sectional), description of the population studied, sample size, type of net evaluated (ITN or LLIN), and outcomes related to *P. falciparum* infection, such as prevalence of infection, incidence, parasitemia, or parasite density, were extracted in the first stage. The second stage involved extracting quantitative meta-analysis data, including the type of net used and specific estimates of *P. falciparum* with their effect sizes. For studies providing raw comparative data, the number of *P. falciparum*-positive cases and the total number of individuals for ITN/LLIN users and non-users were extracted; if any component was unavailable, it was recorded as “Not reported.” If studies presented adjusted effect estimates, these included adjusted odds ratios (AORs), risk ratios, or prevalence ratios, and their 95% confidence intervals were extracted. Cross-checking of all extracted data for accuracy was performed prior to the analysis. Data extraction was performed independently by two reviewers using the standardized template. Any disagreements were resolved by consensus after re-checking the source articles, and the final dataset was verified prior to analysis.

### 2.6. Data Organization

All data were structured systematically in Microsoft Excel to maintain a consistent and clean data format. As the studies provided outcomes in two formats (raw counts and adjusted effect estimates), the dataset was split into two groups:

Group 1: 6 studies reporting raw counts of Plasmodium falciparum infection among ITN/LLIN users and non-users. These data were organized into the following columns: Study ID, Country, Study Design, Population, Population Type, Type of Net, Total Sample Size, Pf-positive users, Total users, Pf-positive non-users, Total non-users, and Crude OR. These variables enabled the computation and synthesis of crude odds ratios in Meta-analysis 1.

Group 2: 7 studies (9 comparisons) reporting adjusted effect measures, and the data were organized into columns: Study ID, Country, Study Design, Population, Population Type, Type of Net, Total Sample Size, Effect Type, Adjusted Effect Estimate, and Adjusted 95% CI. These studies formed the dataset for Meta-analysis 2.

Adjusted prevalence ratios (PRs) were extracted where reported; however, they were not included in the quantitative synthesis. Although PRs are appropriate for cross-sectional studies, they are not directly comparable with odds ratios and cannot be estimated for case–control designs, which constituted part of the included evidence. Recalculation of PRs would have required additional assumptions and transformation of adjusted estimates, potentially introducing bias. Therefore, to maintain methodological comparability across mixed study designs and effect measures, only odds ratios were synthesized. As only one eligible study reported a PR, it was excluded from the pooled analysis.

### 2.7. Risk of Bias Assessment

The risk of bias was evaluated using a structured, step-by-step approach to assess the methodological rigor of the included studies. In the first step, the design of each study was ascertained to apply the corresponding critical appraisal tool: the AXIS tool for cross-sectional studies [18], Newcastle–Ottawa Scale (NOS) for case–control studies [19], and Cochrane RoB 2.0 tool for cluster randomized trials [20]. Using the chosen tool, domain-level information on the potential risk of bias was systematically extracted from each included study, including sampling method, validity of measurement, data analysis and statistical reporting, internal consistency, selection process/criteria, exposure assessment, outcome measure, and transparency regarding ethical approval and funding. Each domain was subsequently judged to be at low, some concerns, or high risk based on the criteria of the respective tool. Finally, a judgment on the overall risk of bias was made for each study by combining domain-level ratings and assessing whether methodological limitations affected the validity of the reported outcomes. This procedure also helped ensure a uniform, valid review of study quality across all eligible designs.

### 2.8. Statistical Analysis

All statistical analyses were conducted in R software (version 4.4.2; R Foundation for Statistical Computing, Vienna, Austria) using the packages meta [21], metafor [22], and readxl [23]. As two types of effect measures were reported in the included studies, two separate meta-analyses were conducted. ITNs and LLINs were initially analyzed as a combined exposure to maximize statistical power and reflect how these interventions are frequently reported in epidemiological studies; where data permitted, stratified subgroup analyses by net type (ITN vs. LLIN) were conducted to explore heterogeneity. Meta-analysis 1 included studies that reported raw 2 × 2 counts of *P. falciparum* infection prevalence among ITN/LLIN users and non-users. The crude odds ratios (ORs) were estimated using the metabin () function and combined using a random-effects model with the DerSimonian–Laird estimator. This analysis further involved forest plot generation, heterogeneity measurement (Q-test, I^2^, τ^2^) [24], assessment of publication bias using funnel plots and Egger’s linear regression test [25], and the trim and fill method [26]. Sensitivity analysis was conducted using leave-one-out (LOO) influence diagnostics [27], and potential sources of heterogeneity were investigated using meta-regression models with population type and study design as moderators [28]. Subgroup analysis was then carried out to compare the effects of ITN and LLIN interventions. Meta-analysis 2 synthesized adjusted effect sizes and was performed for studies that provided adjusted odds ratios (aORs) with their respective 95% confidence intervals (CIs). A random-effects model was conducted using the Hartung–Knapp adjustment in the metafor package. This study included forest plots, evaluation of heterogeneity (Q-test), assessment of publication bias using funnel plots, and Egger’s regression test. A leave-one-out sensitivity analysis was also performed. Meta-regression was performed to explore country and population type as moderators, and subgroup analysis was carried out by net type (ITN vs. LLIN). All tests were two-sided, and statistical significance was defined at *p* < 0.05.

## 3. Results

### 3.1. Study Selection

**Figure 1:**
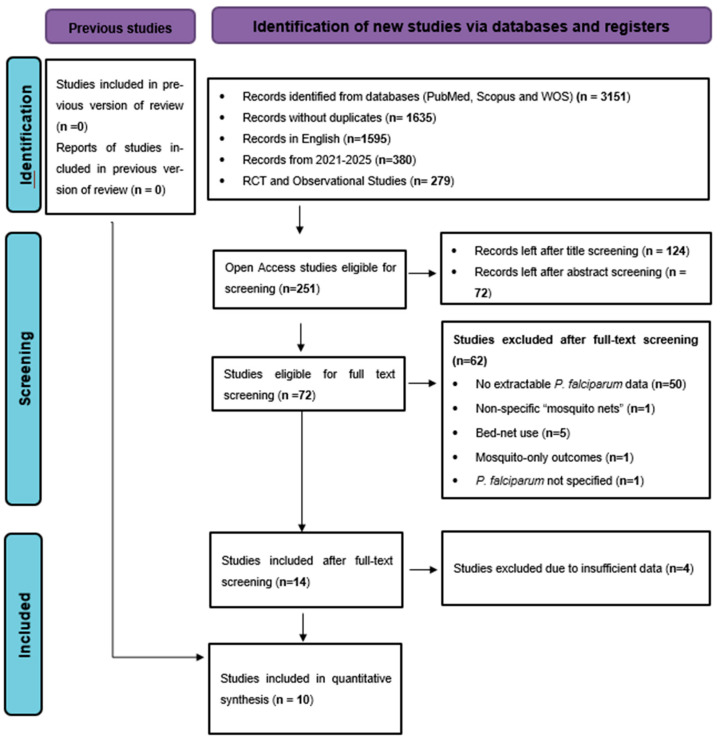
PRISMA flow diagram illustrating the identification, screening, eligibility assessment, and final inclusion of studies in the quantitative meta-analysis.

The study selection process followed PRISMA 2020 guidelines to ensure transparent and systematic identification of eligible studies. The initial search across PubMed, Scopus, and Web of Science yielded 3151 records (PubMed = 848; Scopus = 1203; WOS = 1100). After removing 1516 duplicates, 1635 unique articles remained. Of these, 1595 were published in English, and limiting the search to studies published from 2021 to 2025 reduced the total to 380 records. Applying study design filters to retain only randomized controlled trials and observational epidemiological studies resulted in 279 articles, and restricting to open-access publications left 251 records for title screening. Based on title relevance, 124 studies proceeded to abstract screening, of which 72 met the criteria for full-text evaluation. During full-text screening, 62 articles were excluded, including 50 studies that lacked extractable numerical data on *Plasmodium falciparum* infection prevalence outcomes by ITN/LLIN exposure [29,30,31,32,33,34,35,36,37,38,39,40,41,42,43,44,45,46,47,48,49,50,51,52,53,54,55,56,57,58,59,60,61,62,63,64,65,66,67,68,69,70,71,72,73,74,75,76,77,78], 1 study referring only to “mosquito nets” without specifying insecticide treatment [79], 5 studies reporting bed-net utilization without distinguishing ITNs/LLINs [80,81,82,83,84], 1 entomological study reporting mosquito-only infection outcomes [85], 1 study that did not specify *P. falciparum* [86], and 4 studies with insufficient statistical data [87,88,89,90]. After applying all eligibility criteria, 10 studies fulfilled the requirements and were included in the final quantitative meta-analysis. The complete selection process is depicted in the PRISMA flow diagram (Figure 1).

### 3.2. Study Characteristics

The study characteristics table is an aggregated description of all included studies and presents important elements of study methods, including study design, population demographics, sample size details, the type of insecticide-treated net examined, and *P. falciparum*-related outcomes. Such a structured presentation facilitates comparisons between studies and helps interpret the heterogeneity of effect estimates. These key features are described in Table 1 and provide readers with a snapshot of the evidence base supporting the pooled analyses performed for this review.

### 3.3. Risk of Bias Assessment

**Figure 2:**
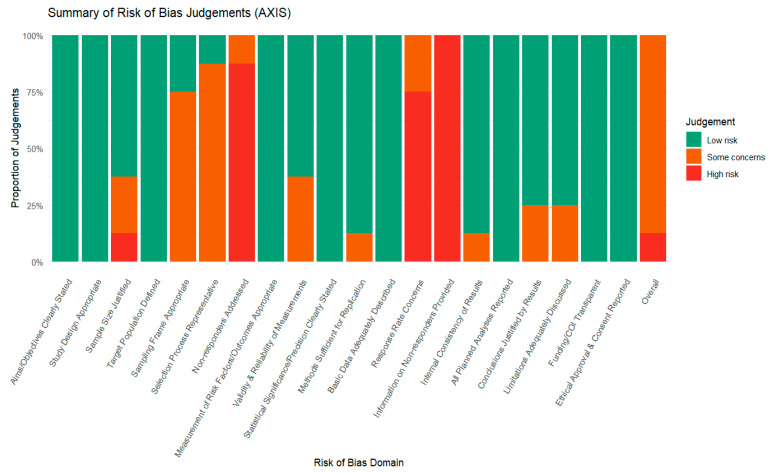
Summary of risk of bias assessments for cross-sectional studies using the AXIS tool, showing the proportion of judgments rated as low risk, some concerns, or high risk across all evaluated domains.

**Figure 3:**
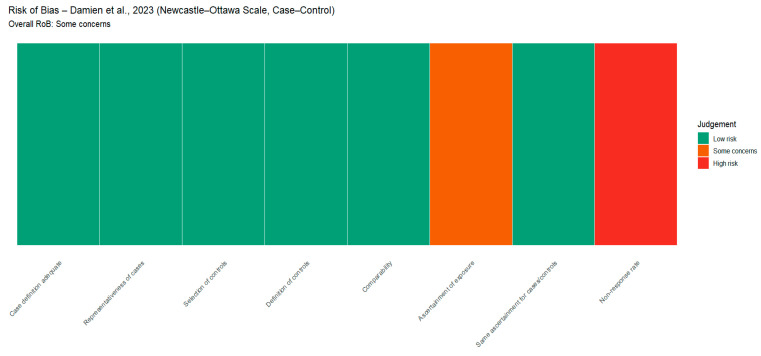
Risk of bias assessment for Damien, Kesteman [15] using the Newcastle–Ottawa Scale for case–control studies, showing judgments across key domains and the overall rating of “some concerns”.

**Figure 4:**
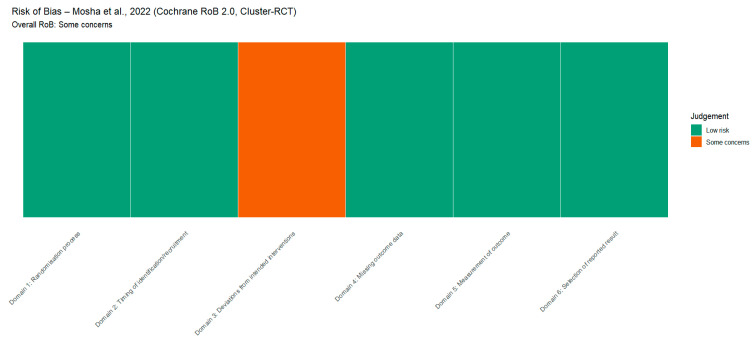
Risk of bias assessment for Mosha, Kulkarni [96] using the Cochrane RoB 2.0 tool for cluster-randomized trials, showing domain-level judgments and an overall rating of “some concerns”.

The risk of bias (RoB) was evaluated for the included studies using relevant tools for the study type. Cross-sectional studies were assessed using the AXIS tool, case–control studies using the Newcastle–Ottawa Scale (NOS), and cluster-randomized trials using the Cochrane Risk of Bias 2.0 (RoB 2.0) framework. Evaluation of methodological components, such as the quality of the study design, representativeness of the sample, validity and reliability of measurement, non-response and missing-data adjustment, comparability between groups, and transparency in reporting, is assessed using these instruments. Figure 2, Figure 3 and Figure 4 visually summarize the domain-level risk of bias assessments and study-level risk of bias across domains, depicting the percentage of studies judged as low, some concerns, or high risk in each domain.

### 3.4. Crude Odds Ratios (Unadjusted Effect Estimates)

**Figure 5:**
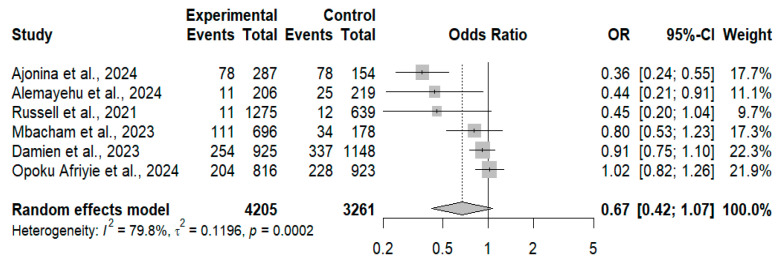
Forest plot of crude odds ratios comparing *Plasmodium falciparum* infection among ITN/LLIN users versus non-users, showing study-level estimates, pooled random-effects result, and heterogeneity statistics [91,92,15,95,98,99].

Six studies involving 7466 participants (4205 ITN/LLIN non-users and 3261 users) were included in the meta-analysis of crude effect estimates. The pooled random-effects analysis yielded an odds ratio of 0.67 (95% CI: 0.42–1.07; *p* = 0.078), indicating no statistically significant association between ITN/LLIN use and *Plasmodium falciparum* infection prevalence (Figure 5). Substantial between-study heterogeneity was observed (I^2^ = 79.8%, τ^2^ = 0.1196, *p* = 0.0002). To explore potential sources of heterogeneity, meta-regression analyses were performed. Study design explained 36.1% of the between-study heterogeneity, although the moderator effect was not statistically significant (*p* = 0.166). Population type accounted for a larger proportion of heterogeneity (59.3%), with borderline statistical significance (*p* = 0.059). Subgroup analyses stratified by net type yielded pooled odds ratios below unity for both ITNs (OR = 0.72) and LLINs (OR = 0.59); however, neither subgroup demonstrated a statistically significant association, and no significant difference was observed between net types (*p* = 0.677).

**Figure 6:**
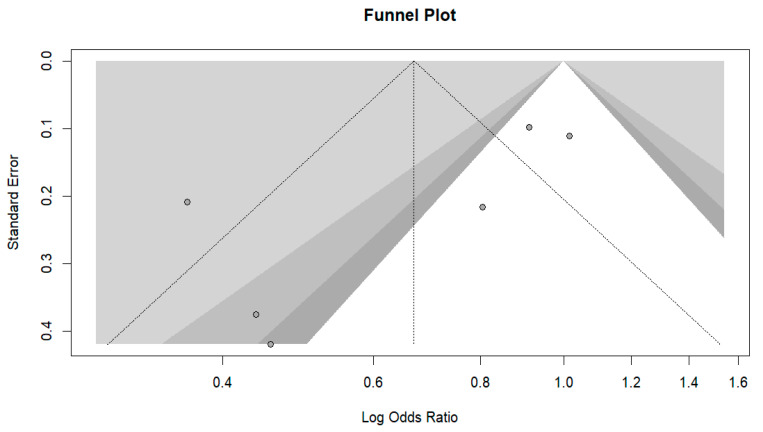
Funnel plot assessing publication bias for crude-effect studies, showing the distribution of log odds ratios against standard error with confidence region shading.

**Figure 7:**
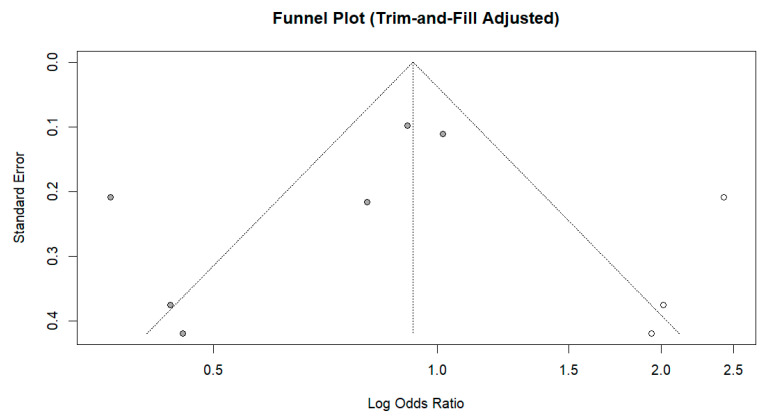
Trim-and-fill–adjusted funnel plot for crude-effect studies, illustrating imputed symmetry and the adjusted distribution of log odds ratios against standard error.

Sensitivity analysis with leave-one-out diagnostics indicated that the pooled effect size was constant in direction and magnitude when each study was omitted, suggesting that no single study substantially affected this meta-analysis. However, heterogeneity varied across iterations—dropping to 41.7% when Ajonina, Ajonina-Ekoti [91] was excluded and increasing to over 78% when Mbacham, Mosume [95] or Damien, Kesteman [15] were excluded—the overall effect continued to favor ITN/LLIN use. The funnel plot (Figure 6) showed mild asymmetry, and Egger’s regression was borderline statistically significant (*p* = 0.090), suggesting evidence of small-study effects. When the trim-and-fill method was used, three missing studies were added on the right side of the funnel plot, and the pooled estimate was pulled toward the null (adjusted OR = 0.93, 95% CI: 0.55–1.57) (Figure 7); however, the associations remained statistically nonsignificant. Overall, the aggregate data do not suggest marked publication bias, and the central pooled effect estimate appears resistant to it.

### 3.5. Adjusted Odds Ratios (Adjusted Effect Estimates)

**Figure 8:**
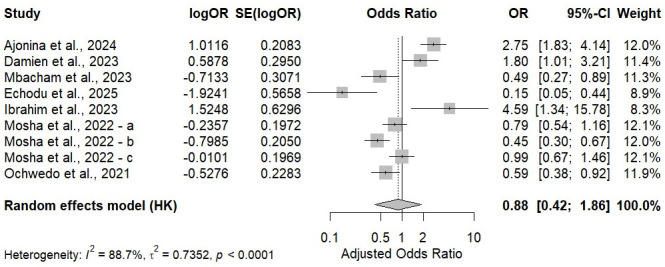
Forest plot of adjusted odds ratios for *Plasmodium falciparum* infection comparing ITN/LLIN users with non-users, showing study-level estimates, pooled Hartung–Knapp random-effects result, and heterogeneity statistics [91,15,93,94,95,96,97].

A meta-analysis of adjusted effects, comprising nine studies, was performed across populations (pregnant women, children, adults, and all-age community members) in six African countries. The combined random-effects model revealed no statistically significant difference in the odds of Plasmodium falciparum infection prevalence among ITN/LLIN users versus non-users after controlling for confounders (aOR = 0.88; 95% CI: 0.42–1.86; *p* = 0.71). There was considerable heterogeneity (I^2^ = 88.7%, τ^2^ = 0.7352; *p* < 0.0001), suggesting significant diversity in adjusted effects across studies (Figure 8). Meta-regression analyses were conducted to investigate the sources of this heterogeneity. The country-level meta-regression accounted for 28.2% of the heterogeneity, mainly attributable to significantly lower adjusted odds in Uganda (*p* = 0.0378), which affected the effect size but not in any other country. The population-type meta-regression explained 27.4% of the heterogeneity, demonstrating that the infection risk was highly variable across age groups. Children under 5 years showed a stronger association (estimate = –3.45; *p* = 0.0098), but there were no statistically significant moderators among older age groups. Significant heterogeneity was also observed in the subgroup analysis by net type. Studies evaluating ITNs yielded a pooled adjusted odds ratio of 0.29 (95% CI: 0.0001–587.01; I^2^ = 71.7%), while LLIN-only studies produced pooled estimates closer to the null (aOR = 1.15; 95% CI: 0.55–2.42; I^2^ = 89.2%). A statistically significant difference was observed between subgroups (Q = 4.15; *p* = 0.0415), indicating that net type contributed to between-study heterogeneity; however, the wide confidence intervals indicate substantial uncertainty in subgroup-specific estimates.

**Figure 9:**
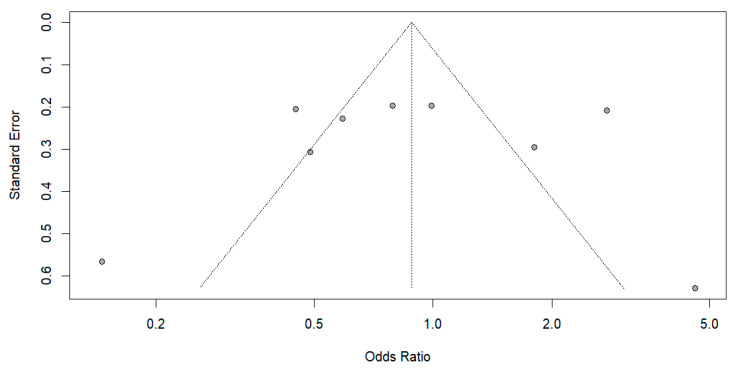
Funnel plot assessing publication bias for adjusted-effect studies, showing the distribution of odds ratios against standard error.

Sensitivity analyses revealed that the pooled effect estimate was generally robust across all leave-one-out iterations, and none of the excluded studies substantially changed the direction or magnitude of the overall correlation (from –0.2836 to 0.0356). While heterogeneity was uniformly high across all iterations (I^2^ 89–93%), excluding individual studies did not alter the effect towards significance, thus confirming that a specific study did not exert undue influence on the adjusted-effect meta-analysis. The results of the visual assessment showed that the funnel plot was symmetrical (Figure 9), and Egger’s test did not show statistically significant small-study effects (t = −0.11; *p* = 0.913).

## 4. Discussion

This meta-analysis consolidated recent epidemiological evidence (2021–2025) on the association between ITNs/LLINs and *P. falciparum* infection prevalence, showing no statistically significant pooled association, with substantial uncertainty across studies. The crude-effect meta-analysis indicated that ITN/LLIN users had lower pooled odds that did not reach statistical significance (OR 0.67, 95% CI: 0.42–1.07), whereas the adjusted-effect meta-analysis yielded an aOR of 0.88 (95% CI: 0.42–1.86), indicating attenuation of the pooled estimate after adjustment for confounding. The observed attenuation of effect estimates after adjustment highlights the influence of confounding factors on the association between ITN/LLIN use and *P. falciparum* infection prevalence. In observational epidemiological studies, net use is strongly associated with socioeconomic status, housing quality, education, urban–rural residence, access to healthcare, and co-interventions, all of which independently affect malaria risk. The weakening of associations after adjustment suggests that residual or imperfectly measured confounding is likely to contribute to the apparent associations observed in crude analyses, thereby limiting causal interpretation. Both analyses demonstrated significant heterogeneity, but meta-regression and subgroup analyses identified important sources of variability including effect modification by age group (children < 5 years), country-level differences (notably Uganda), and net type, rather than consistent protective effects. Taken together, these results indicate that insecticide-treated nets remain an important tool for malaria control, although their association with *P. falciparum* infection varies considerably across contemporary African settings.

Yang, Kim [7] reported consistently strong and statistically significant reductions in malaria risk for both LLINs (OR 0.44) and ITNs (OR 0.59), with LLINs being the more effective product when adjusting for transmission intensity, duration of use, and study design. The most recent pooled estimates, particularly the non-significant adjusted aOR, are weaker than those of this previous work due to differences in methods, but they point to the potential decrease in the effectiveness of nets under current programmatic conditions compared to previous years of widespread large-scale upscaling and lower resistance. Nevertheless, our evidence for stronger protection in high-transmission settings is generally consistent with that of Yang, Kim [7], who found higher efficacy when transmission was high and nets were used longer. At the level of individual studies, several included and comparator studies support a protective role for nets. Atieli, Zhou [100] reported a decrease in parasite prevalence among LLIN users compared with non-users during the rainy season in the highlands of western Kenya, and Larsen, Hutchinson [101] reported lower parasite infection and reduced child mortality among ITN-owning households residing in communities with higher ITN coverage. In contrast, Damien, Kesteman [15] in Benin found no effect or only a light effect of LLINs, including IRS, on infection and uncomplicated clinical malaria compared to the control. The study points to weak pooled effects and suggests that nets alone may not be sufficient in contemporary settings.

The high heterogeneity in crude and adjusted analyses may reflect the intricate and dynamic picture of vector control. Research on next-generation or dual-active-ingredient nets helps interpret this pattern. Epstein, Gonahasa [9] demonstrated that new LLINs in Uganda led to a 23% decrease in malaria incidence during the first 12 months after distribution. However, this reduction was not sustained into the second year, suggesting that the net’s effectiveness declined quickly under routine conditions. Tapsoba, Guelbeogo [102] found that coverage of G2 double-AI Interceptor^®^ nets led to dramatic reductions in mosquito density and transmission indices for the first 2 years post-intervention, before any re-emergence in 3rd year; similar net types treated with pyrethroids returned to pre-control conditions earlier. These results were supported by experiments and durability measurements. Perugini, Pichler [103] reported the combination of (L1014F, L1014S, V402L) harboring with alleles was detected among mosquito species in Burkina Faso representing a direct risk for pyrethroid-based net efficacy; Oyweri, Onyango [11] observed that PBO-LLINs and pyrethroid-only LLINs from western Kenya both lost physical integrity and bio efficacy within 18 months; and Martin, Messenger [104] demonstrated a new type of LLIN (PBO, pyriproxyfen and chlorfenapyr combination) gradually loses bio-efficacy over 3 years despite being superior to standard nets when used on their own. This pattern of results is consistent with the meta-analytic findings of both reduced and variable effects due to biologically and operationally plausible attenuation in a setting of high resistance pressure and rapid net decay.

The findings must also be considered in relation to access, coverage, and actual net usage, which several regional and multi-country studies show are vital inputs for effectiveness. However, despite high ownership in some areas, use may be suboptimal or seasonal. Atieli, Zhou [100] found high ITN ownership. However, overall ITN use in the Kenyan highlands is only 56%, with higher and lower use during the wet and dry seasons, respectively, as measured by thin blood films among school-aged children, which may explain the modest epidemiological impact. Community coverage assays have also demonstrated that the protective efficacy of ITNs is highly dependent on community-level penetration. found that households with ITN ownership living in communities with 30–50% coverage had significantly lower parasite prevalence and all-cause child mortality. However, no apparent “mass effect” was observed among non-ITN-owning households at any level of coverage. It should also be noted that disparities in access would shape the national and regional multipliers on the impact of nets. Inusah, Gbeti [14] found marked by prosperity and residence-related inequalities in household ownership of ITNs/IRS across 34 African countries; strong pro-rich or pro-urban patterns in some cases, and negative patterns (pro-poor or pro-rural) in others. Among countries, pregnant women, and children under 5 years, unique barriers or facilitators may be experienced. For example, Ameyaw, Adde [105] reported higher ITN use among rural and poor pregnant women than among urban women in Nigeria. Konlan, Kossi Vivor [106] conducted a scoping review and reported that hot weather perceived low mosquito density, net properties (color, odor, shape), and other uses of nets were significant barriers to net use among children under 5 years old. These structural and behavioral factors likely contribute to the attenuation and variability of pooled estimates observed in this meta-analysis.

In this context, the current meta-analysis contributes by considering the most recent (2021–2025) human epidemiological studies and performing separate analyses of crude and adjusted effect estimates. This suggests that confounding adjustment (particularly for socioeconomic status, housing conditions, and co-interventions) could reduce the perceived net effects. It is also a valuable adjunct to extensive multi-country analyses, such as those by Fullman, Burstein [107] and Damien, Kesteman [15], which investigated ITNs and IRS under normal conditions, finding mostly setting-specific or minor effects on parasitemia and no effect on child mortality. However, some limitations need to be considered in future studies. First, the number of studies included in each pooled estimate (particularly those adjusted effects and subgroups) was relatively small compared with previous worldwide meta-analyses, which may weaken statistical power and precision. Second, substantial heterogeneity remained across analyses and was only partially explained despite meta-regression by population type, country, and study design, suggesting important residual and largely unmeasured sources of variability (vector species composition, resistance intensity, net age, co-coverage with IRS or chemoprevention, and health system performance). This high and largely unexplained heterogeneity strongly limits the interpretability and generalizability of the pooled estimates, and summary effect sizes should therefore be interpreted with caution. Third, because only open-access, English-language sources were used, relevant studies may have been excluded, leading to a biased sample. Finally, the outcomes of this meta-analysis were limited to *P. falciparum* infections and did not reflect clinical malaria or mortality. While this is consistent with most recent studies, direct comparisons with earlier studies, including broader morbidity and mortality endpoints, are limited. Given the predominance of observational studies, the findings of this meta-analysis should be interpreted as epidemiological associations rather than causal effects, particularly when considering policy or programmatic implications. Comparisons between ITNs and LLINs should be interpreted with caution, as they were based on a small number of studies and, in the case of ITNs, were characterized by extremely wide confidence intervals. These subgroup analyses were therefore exploratory in nature and not designed to establish differences in effectiveness between net types. Observed between-group differences should be interpreted as sources of heterogeneity and hypothesis-generating rather than confirmatory findings.

The evidence suggests that while ITNs and LLINs continue to affect *P. falciparum* infection, their association with *P. falciparum* infection prevalence in programmatic settings is inconsistent and highly context dependent. Studies that combined ITNs and IRS [15,107] and evaluated dual-AI or next-generation LLINs [9,102,104] indicate that integrated approaches, along with better products, may improve impact, especially in high-transmission, high-resistance settings, although these benefits could be short-lived as nets age and resistance increases [11,103]. Meanwhile, continued inequities in access [14] and gaps in ownership versus usage [100,106], as well as urban-versus-rural differences in use [105], highlight the importance of context-relevant delivery strategies, ongoing behavior change communication, and net durability/user preference considerations. In the future, comparative epidemiological studies should report crude and adjusted estimates, stratify by age group and transmission setting, and include collected net data (type of net, age of net, and integrity of net) along with co-interventions. Such standardized reporting will be critical for updating pooled estimates, deepening understanding of effect modifiers, and guiding the rational use of next-generation ITNs in larger integrated malaria control and elimination programs.

## 5. Conclusions

The meta-analysis demonstrates that ITNs/LLINs do not show a statistically significant pooled association but exhibit substantial variability in their association with *P. falciparum* infection prevalence across a range of epidemiological contexts. Although the combined crude and adjusted effect estimates were not statistically significant, the pooled estimates were consistently below unity, indicating directional but inconclusive associations. There was substantial heterogeneity across studies, likely due to variations in study design, population type, and country. Subgroup and meta-regression analyses also indicated that the associations between ITN/LLIN use, and infection risk are context-specific, with greater variability observed across countries, age groups (particularly children under 5 years), and net types, rather than consistent evidence of differential protection. Nevertheless, rising pyrethroid resistance, loss of net physical integrity, reduced bio-efficacy over time, and persistent net ownership-to-use gaps continue to be significant barriers to sustainable impact. These results emphasize that while ITNs/LLINs are a cornerstone of malaria prevention, their epidemiological impact on *P. falciparum* infection may be limited or inconsistent when implemented as the sole intervention, particularly in high-resistance or low-use settings. Enhanced malaria control thus requires combining next-generation or dual-active-ingredient nets with improved population-level coverage and adherence, and complementing net use with other interventions, such as indoor residual spraying or targeted chemoprevention. Further studies should focus on standardized reporting of effect estimates, rigorous evaluation of new net technologies, and context-specific implementation options to maximize the contribution of ITNs/LLINs towards global targets for malaria elimination.

## Figures and Tables

**Table 1:** Study Characteristics For Included Studies.

Study ID	Country	Study Design	Population	Sample Size	Intervention (ITN/LLIN)	Outcomes Reported
Ajonina, Ajonina-Ekoti [91]	Cameroon	Cross-sectional study	Pregnant women attending ANC who owned LLIN	441	LLIN	*P. falciparum* infection prevalence; Parasitemia prevalence
Alemayehu, Abossie [92]	Ethiopia	Cross-sectional study	Pregnant women attending ANC clinics in Majang Zone	425	ITN	*P. falciparum* infection prevalence; Parasitemia prevalence; Parasite density
Damien, Kesteman [15]	Benin	Case–control study (nested in cross-sectional survey)	General population (all ages) from households in Djougou and Cobly	4850	LLIN use every night vs. less/none; comparisons: LLIN only, IRS only, LLIN + IRS, no LLIN/IRS	*P. falciparum* infection prevalence
Echodu, Ajolorwot [93]	Uganda	Cross-sectional study	Children <5 years old from selected households in northern districts	597	ITNs: ownership 77.6%, ~65% impregnated nets; 48.7% used previous night	*P. falciparum* infection prevalence; Parasitemia prevalence
Ibrahim, Bello [94]	Nigeria	Cross-sectional (hospital-based)	Apparently healthy adults ≥40 years attending free health programmes	232	LLIN (sleeping under LLIN vs. not)	*P. falciparum* infection prevalence; Parasite density
Mbacham, Mosume [95]	Cameroon	Cross-sectional (prospective)	Pregnant women ≥36 weeks attending ANC in Mount Cameroon area	874	LLIN/ITN; high ownership (~80%) and usage (~60%)	*P. falciparum* infection prevalence (microscopic and sub-microscopic); Parasitemia prevalence; Parasite density
Mosha, Kulkarni [96]	Tanzania	Cluster-RCT	Children aged 6 months–14 years in Misungwi district	Cross-sectional: 4403 at baseline and 14,163 at 12–24 months; Cohorts: 6353	Four LLIN types: pyrethroid-only, pyriproxyfen LLIN, chlorfenapyr LLIN, PBO LLIN	*P. falciparum* infection prevalence; Infection incidence
Ochwedo, Omondi [97]	Kenya	Cross-sectional study	Patients seeking malaria treatment (all ages, including pregnant women)	367	LLIN	*P. falciparum* infection prevalence; Parasitemia prevalence; Parasite density
Opoku Afriyie, Antwi [98]	Ghana	Cross-sectional study	Suspected malaria patients attending AGH and MGH	1739	ITN (ownership and usage; slept under ITN previous night)	*P. falciparum* infection prevalence
Russell, Grignard [99]	Solomon Islands	Cross-sectional study	Residents ≥ 5 years in selected villages across provinces	1977	ITN (predominantly LLINs; use the previous night)	*P. falciparum* infection prevale

## Data Availability

The original contributions presented in this study are included in the article. Further inquiries can be directed to the corresponding author.
